# Physician-Patient Communication and Physicians’ Acceptance of a Tailored Digital Health Information Service: Quantitative Online Survey

**DOI:** 10.2196/80620

**Published:** 2026-03-06

**Authors:** Paula Memenga, Elena Link

**Affiliations:** 1 Department of Journalism and Communication Research, Hanover University of Music Drama and Media, Expo Plaza 12, Hannover, 30539, Germany; 2 Department of Communication, Johannes Gutenberg University Mainz, Mainz, Germany

**Keywords:** digital health information, information provision, physician-patient communication, health technology acceptance, UTAUT2, extended unified theory of acceptance and use of technology

## Abstract

**Background:**

Providing tailored information is an essential part of health care. However, physicians often lack time for detailed education during the consultation. An additional, tailored digital health information service (DHIS) could help physicians meet their patients’ information needs regardless of time and place and extend physician-patient communication to the digital realm.

**Objective:**

This study aimed to examine physicians’ intentions to provide a DHIS to their patients and identify facilitating factors and barriers, guided by the extended unified theory of acceptance and use of technology.

**Methods:**

A cross-sectional online survey with German physicians from various specialties was conducted in March 2022. The sample (N=364) ranged in age from 33 to 75 years (mean 53.92, SD 8.12), and the majority were male participants (31.9% [n=116] female participants). A blockwise multiple linear regression analysis was conducted to identify facilitating factors and barriers of physicians’ intentions to provide a DHIS.

**Results:**

Overall, 54.1% (n=197) of the surveyed physicians were (rather) willing to provide a tailored DHIS, 23.9% (n=87) were undecided, and 22% (n=80) were (rather) not willing to provide such a service to their patients. The overall model of a blockwise multiple linear regression analysis explained 56.8% of the variance of physicians’ intentions. Perceived usefulness for job performance and patient outcomes as well as personal innovativeness was positively associated with physicians’ intentions to provide a DHIS to their patients. Ease of use, social influence, facilitating conditions, price value, and habit were not associated with their intentions.

**Conclusions:**

The perspective of the majority of surveyed physicians suggests that a tailored DHIS seems to be a promising way to provide additional health information and thus enhance face-to-face physician-patient communication. Efforts supporting the implementation of DHIS should address job performance and patient outcomes in particular. Further, physicians with a more positive attitude could serve as multipliers to increase the adoption of DHIS.

## Introduction

### Digital Provision of Health Information

Since patients have a right to informed medical decision-making [1], and providing tailored information is an essential part of health care [2], physicians are required to meet their patients’ needs for adequate, high-quality information [3]. However, physicians often lack the time to provide their patients with detailed information during the consultation [4,5]. Further, an increasing amount of false or misleading information, particularly prevalent during the COVID-19 pandemic [6], poses a challenge in providing accurate health care. These barriers emphasize the need to rethink physician-patient communication in general and consider new ways of providing high-quality health information to patients.

The digital age, characterized by advances in information and communication technology [7,8], offers new opportunities for information provision that could help to supplement the traditional relationship between health professionals and their patients [9]. For example, face-to-face physician-patient communication could be extended to the digital provision of health information, enabling physicians to meet their patients’ needs for high-quality information regardless of time and place. Such a digital health information service (DHIS) is also in line with global objectives of strengthening health care and improving patient education through the implementation of digital health technologies [10,11]. It can be understood as a web-based platform through which physicians can provide their patients with information tailored to their individual disease or health situation (eg, on symptoms, medical examinations, treatment options, prevention, and early detection measures) in addition to the explanations given during the consultation. Thus, patients receive tailored, high-quality information from their most trusted source of health information [12,13] that can be accessed via a computer or mobile phone, enabling them to autonomously build an adequate knowledge base for informed decision-making.

A basic requirement for realizing the potential of a DHIS in overcoming barriers to information provision and complementing routine health care is physicians’ acceptance of such a service [14,15]. Existing studies on digital health interventions revealed mixed results regarding physicians’ willingness to engage with digital services. On the one hand, physicians in Germany perceived great opportunities from health apps with regard to prevention, reminder, and lifestyle support features [16] and showed high use of internet-related technologies for patient support [17]. On the other hand, prior studies showed a moderate acceptance of mobile apps for disease management [18] and a rather low acceptance of eHealth interventions in inpatient routine care [19].

Based on the heterogeneous state of research, this study aimed to examine physicians’ intentions to provide a DHIS to their patients as well as identify facilitating factors and barriers. The study was guided by the extended unified theory of acceptance and use of technology (UTAUT2) [20], which is a widely accepted theory for understanding physicians’ adoption of digital health interventions [21,22] and informing eHealth implementation [23].

### Theoretical Background

#### Postulates of the UTAUT2

The UTAUT2 [20], which we used as a guiding framework to examine physicians’ intentions to provide a DHIS, is a common technology acceptance model that was originally developed to explain consumers’ acceptance and use of innovative technologies. It can be understood as an integrative model as it unifies elements of 8 theoretically proven models from different fields of research, such as information systems research (eg, Technology Acceptance Model), sociology (eg, Innovation Diffusion Theory), and psychology (eg, theory of planned behavior). UTAUT2 is an extension of the unified theory of acceptance and use of technology (UTAUT) [24], which postulates performance expectancy, effort expectancy, social influence, and facilitating conditions as key determinants of usage intentions. UTAUT2 additionally considers hedonic motivation, price value, and habit as predictors of the intention to use a specific technology.

Previous research has shown that UTAUT and UTAUT2 are also effective in various health contexts, explaining patients’ and health care providers’ acceptance of health-related technologies such as teleconsultation technology [25-27], electronic health records [28-32], mobile health apps [18,33-37], or eHealth interventions [19,38].

#### Modeling Predictors of Physicians’ Intentions to Provide a DHIS Using UTAUT2

##### Overview

Based on the UTAUT2 and extant research in the health context, performance expectancy, effort expectancy, social influence, facilitating conditions, price value, and habit were integrated into our research model. Hedonic motivation is conceptualized within UTAUT2 as the pleasure or enjoyment derived from technology use and has primarily been applied in consumer-oriented and leisure-related contexts [20]. In contrast, physicians’ use of digital health technologies occurs within professional roles and clinical responsibilities. Consistent with prior health care-related applications of UTAUT2, we therefore excluded hedonic motivation [27,31,33]. In addition, personal innovativeness was added as an important factor to explain physicians’ intentions to provide a DHIS to their patients.

##### Performance Expectancy

In the health context, performance expectancy [20,24] is defined as the degree to which a physician expects that using a specific digital technology provides personal benefits in the daily work life, for example saving time during office hours or improving patient communication [22]. It thus describes the perceived usefulness of the DHIS for improving physicians’ job performance. Previous research has shown that performance expectancy is a key predictor of healthcare providers’ intention to use digital, health-related technologies [18,19,28]. However, digital health services for tailored information provision not only aim to improve job performance but also to promote patient-centered care [10]. In line with research showing that physicians perceive digital health services’ potential to support patients’ health promotion and prevention [16,17], performance expectancy might also refer to the physicians’ perceived usefulness for improving patient outcomes, for example, meeting information needs, supporting decision-making, and improving health and well-being. According to the theoretical assumptions and state of research outlined, we derived the first hypothesis:

*H1****:*** *Perceived usefulness for job performance (H1a) and perceived usefulness for improving patient outcomes (H1b) will be positively related to physicians’ intention to provide a DHIS*.

##### Effort Expectancy

Effort expectancy [20,24] refers to the perceived ease of use associated with using a specific technology which is a well-supported facilitator of a higher intention to use a digital, health-related technology [25,28,29,37]. In the context under study, effort expectancy refers to the extent to which a physician expects to be able to use a DHIS with low effort [22]. We derived the following hypothesis:

*H2: Perceived ease of use will be positively related to physicians’ intention to provide a DHIS*.

##### Social Influence

Social influence [20,24] covers subjective norms as a known driver of a variety of social and health behaviors such as using digital, health-related services [19,28,39]. Applied to our study, these codes of conduct can be defined as the extent to which a health care provider perceives that important reference groups think that he or she should use a particular technology [22]. In the medical setting, relevant reference groups could be patients as well as colleagues [19]. Accordingly, the perception that patients think one should provide a tailored DHIS for them and that colleagues think one should provide digital information to one’s patients may be related to the intention to provide such a service. Thus, we assumed the following:

*H3: Subjective norms related to patients (H3a) and colleagues (H3b) will be positively related to physicians’ intention to provide a DHIS*.

##### Facilitating Conditions

Facilitating conditions [20,24] refer to the available external support and resources for using digital technology. For example, the practices’ or hospitals’ technical infrastructure and knowledge necessary to support its implementation could influence physicians’ intentions to provide a DHIS [20,22,24]. In line with former research that found a higher degree of facilitating conditions to be positively related to higher usage intentions among health care providers [25,28,29,39], we derived the following hypothesis:

*H4: Facilitating conditions will be positively related to physicians’ intention to provide a DHIS*.

##### Price Value

Price value [20] refers to the monetary costs associated with using digital technology and positively affects intentions to use digital, health-related technologies in general [26,32,40]. Thus, we assumed the following:

*H5: Price value will be positively related to physicians’ intention to provide a DHIS*.

##### Habit

Habit [20] describes the extent to which the use of digital technology is perceived as an automatic process based on prior experiences which is associated with higher usage intentions [37]. It refers to physicians’ perceptions that they have already developed a routine or habit of using digital technologies such as a DHIS in their daily work lives. Therefore, we postulated the following hypothesis:

*H6: Habit will be positively related to physicians’ intention to provide a DHIS*.

##### Personal Innovativeness

Although personal innovativeness is not included in the UTAUT2 model, first studies support that integrating it into UTAUT2 to explain patients’ and health care providers’ acceptance of digital, health-related technologies provides additional explanatory power [26,33,34,37,41]. Thus, it can be considered as a further important construct that requires more attention in health service acceptance and implementation research [40]. Innovativeness is a personal trait that refers to the “extent to which an individual is innovative by his or her perception of new idea or technology” [26], leading these individuals to be more likely to try innovations. In the current context, it can be postulated that physicians with higher personal innovativeness may be more willing to provide a tailored DHIS for their patients, leading to the following hypothesis:

*H7: Personal innovativeness will be positively related to physicians’ intention to provide a DHIS*.

**Figure 1.**
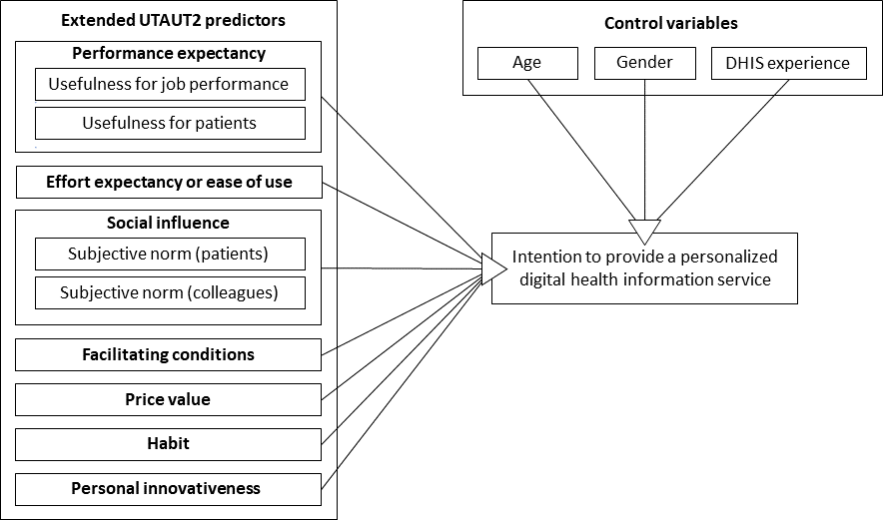
Research model. DHIS: digital health information service; UTAUT2: extended unified theory of acceptance and use of technology.

An overview of the proposed research model is shown in Figure 1.

## Methods

To test our hypotheses, we conducted a cross-sectional online survey in March 2022 with physicians from various specialties (eg, general medicine, cardiology, dermatology, and gynecology). The physicians were recruited and invited to use different strategies. At first, physicians and medical associations were contacted and asked to forward the invitation to the survey. In addition, the call was published in professional journals for physicians. A commercial online access panel was used in the second step.

### Measures

#### Intention to Provide a DHIS

To measure physicians’ intentions to provide a tailored DHIS to their patients, they first received an introduction describing the service as a digital, web-based platform through which tailored health information (eg, on the basics of a disease, symptoms, risks, or treatment options) can be provided in addition to the explanations given during the consultation and accessed by patients at home via a computer or smartphone (S1 in Multimedia Appendix 1). This description was based on the features of an existing DHIS. Subsequently, they were asked whether they had provided a comparable service to their patients in the past and to rate their intentions to (continue to) provide such a service using the 3 original UTAUT2 items adapted to the current context [20] (Table S2 in Multimedia Appendix 1). The 3 items were measured on a 5-point Likert-type scale (1=*strongly disagree* to 5=*strongly agree*). The scale showed excellent internal consistency and was summarized into a mean index (*α*=.94, mean 3.45 [*SD* 1.17]).

#### Performance Expectancy: Perceived Usefulness for Job Performance and Patients

The measurement of perceived usefulness of the DHIS for job performance and improving patient outcomes was adapted from the original UTAUT2 items [20] (Table S2 in Multimedia Appendix 1). Both constructs were measured with 5 items on a 5-point Likert-type scale (1=strongly disagree to 5=strongly agree). The scales showed excellent internal consistency and were summarized into mean indices (usefulness for job performance: *α*=.90, mean 3.49 [SD 0.94]; usefulness for patients: *α*=.91, mean 3.68 [SD 0.83]).

#### Effort Expectancy: Perceived Ease of Use

Perceived ease of use was also measured with a modified version of the UTAUT2 items [20], using 3 items on 5-point Likert-type scales (1=strongly disagree to 5=strongly agree) (Table S2 in Multimedia Appendix 1). The mean index was calculated due to the good internal consistency (*α*=.86, mean 3.21 [SD 1.02]).

#### Social Influence: Subjective Norms

Subjective norms related to patients and colleagues were each measured with 3 items adapted from Venkatesh et al [20] and Park and Smith [42] (Table S2 in Multimedia Appendix 1). The participants assessed their responses on 5-point Likert-type scales, ranging from 1=strongly disagree to 5=strongly agree. Both scales showed acceptable internal consistencies and were transformed into mean indices (subjective norm patients: *α*=.76, mean 2.94 [SD 0.84]; subjective norm colleagues: *α*=.79, mean 2.81 [SD 0.82]).

#### Facilitating Conditions

Facilitating conditions were measured with the original UTAUT2 items [20] (Table S2 in Multimedia Appendix 1). The 4-item measure was rated on a 5-point Likert-type scale (1=strongly disagree to 5=strongly agree). A mean index of high reliability was calculated (*α*=.74, mean 3.50 [SD 0.90]).

#### Price Value

Price value was measured with 3 items about physicians’ requirements for the costs and incentives that might be associated with providing a digital service [20,40] (Table S2 in Multimedia Appendix 1). Participants rated their agreement that a DHIS should be free to physicians and patients on a 5-point Likert-type scale ranging from 1=strongly disagree to 5=strongly agree. However, as the scale showed poor internal consistency (*α*=.47), no mean index could be computed. Therefore, 1 single item that most accurately represented costs for the physician was selected for the analysis (mean 4.28 [SD 0.92]). We note that this item reflects physicians’ perception of whether the service should be free rather than the traditional UTAUT2 definition of price value, which captures the trade-off between perceived benefits and costs of using a technology.

#### Habit

The habit of using digital technologies was measured using the original 3-item UTAUT2 measure [20] (Table S2 in Multimedia Appendix 1) and was evaluated on a 5-point Likert-type scale (1=strongly disagree to 5=strongly agree). The scale showed good internal consistency and was summarized into a mean index (*α*=.81, mean 3.78 [SD 0.95]).

#### Personal Innovativeness

Personal innovativeness was measured with 3 items that were adapted from Baudier et al [27] (Table S2 in Multimedia Appendix 1). Participants rated their responses on a 5-point Likert-type scale, ranging from 1=strongly disagree to 5=strongly agree. The scale was compressed into a mean index (*α*=.84, mean 3.30 [SD 1.03]).

#### Control Variables: Age, Gender, and DHIS Experience

Age, gender, and DHIS experience were used as control variables as these constructs are also assumed to affect technology usage intentions [20]. To measure physicians’ experience with a tailored DHIS, they were asked if they had provided a comparable service to their patients in the past (no/yes; 15.4% (56/364) with DHIS experience).

### Data Analysis

To identify facilitating factors and barriers of physicians’ intentions to provide a tailored DHIS in line with hypotheses 1 to 7, we conducted a blockwise multiple linear regression analysis. The first block considered the control variables age, gender, and DHIS experience. In the second block, the theoretically derived factors were included.

### Ethical Considerations

The study received ethical approval from the Central Ethics Committee at Leibniz University Hannover (review number: EV LUH 19/2021). All participants were informed about the content of the survey prior to their participation and provided informed consent. No personally identifiable information was collected. Participants recruited through the commercial online access panel received financial compensation from the market research company in accordance with its standard remuneration rates. There were no further compensations. All methods were performed in accordance with the Declaration of Helsinki. The study was reported in accordance with the American Psychological Association Journal Article Reporting Standards for quantitative research.

## Results

The sample (N=364) ranged in age from 33 to 75 years (mean 53.92, SD 8.12) and the majority were male participants (31.9% [n=116] female participants). Overall, 97% (n=353) worked in a physician’s practice and 3% (n=11) worked in a hospital or other medical facility in Germany. The majority participated via the commercial online access panel (n=329).

Overall, 54.1% (n=197) of the surveyed physicians were (rather) willing to provide a tailored DHIS, 23.9% (n=87) were undecided, and 22% (n=80) were (rather) not willing to provide such a service to their patients. The overall model of the blockwise multiple linear regression analysis explained 56.8% of the variance of the intention to provide patients with a tailored DHIS. The amount of explained variance of the theoretically derived predictors usefulness for job performance, usefulness for patients, ease of use, subjective norms, facilitating conditions, price value, habit, and personal innovativeness was 42.1%. The results are displayed in Table 1.

**Table 1.** Predictors of physicians’ intentions to provide a digital health information service (N=364)^a^.

Predictors	Unstandardized coefficient (B)	95% CI for B	SE B	*β*	*R*²_corr_	Δ*R²_corr_*
Constant	−0.34	−1.25 to 0.57	0.46			
Sociodemographics and DHIS^b^ experience					0.147	0.147^c^
Age	0.00	−0.01 to 0.01	0.01	−0.02		
Gender (ref. female)	0.00	−0.18 to 0.18	0.09	0.00		
Current use (ref. yes)	0.56	0.33 to 0.80	0.12	0.17^c^		
Extended UTAUT2^d^ predictors					0.568	0.421^c^
Usefulness for job performance	0.36	0.20 to 0.51	0.08	0.29^c^		
Usefulness for patients	0.28	0.10 to 0.46	0.09	0.20^e^		
Ease of use	0.08	−0.04 to 0.19	0.06	0.07		
Subjective norm (patients)	0.04	−0.09 to 0.18	0.07	0.05		
Subjective norm (colleagues)	−0.01	−0.13 to 0.11	0.06	−0.01		
Facilitating conditions	−0.02	−0.14 to 0.11	0.06	−0.01		
Price value	0.03	−0.06 to 0.13	0.05	0.03		
Habit	0.06	−0.06 to 0.18	0.06	0.05		
Personal innovativeness	0.28	0.17 to 0.40	0.06	0.25^c^		

a The table shows model 2 of the blockwise multiple linear regression analysis, identifying physicians’ intentions of providing a tailored digital health information service to their patients. The overall model was significant (*F*_12,350_=40.70, *P*<.001) and explained 56.8% of variance. All variance inflation factors <5.

b DHIS: digital health information service.

c *P*≤.001.

d UTAUT2: extended unified theory of acceptance and use of technology.

e *P *≤.01

As assumed in H1, perceived usefulness for job performance (β=.29; *P*<.001) and perceived usefulness for patient outcomes (*β*=.20; *P*=.002) were positively related to physicians’ intentions to provide a DHIS. Physicians who expect a tailored DHIS to be useful in their daily work life (eg, for saving time during office hours, improving patient communication) and for improving patient outcomes (eg, decision-making, empowerment, improving health) tend to be more willing to provide such a service to their patients. Thus, our results supported H1a and H1b.

H2 focused on perceived ease of using a DHIS, which we postulated to be positively related to physicians’ intentions to provide such a service. However, ease of use (β=.07; *P*=.20) showed no significant association with providing intentions. Based on these findings, H2 had to be rejected.

Considering social influence, we postulated that subjective norms related to patients (H3a) and colleagues (H3b) as physicians’ relevant reference groups are positively related to physicians’ intentions to provide a DHIS. Contrary to our assumptions, both patient-related (β=.03; *P*=.53) and colleague-related subjective norms (β=−.01; *P*=.88) were not associated with physicians’ intentions. Accordingly, H3a and H3b had to be rejected.

In H4, we assumed that facilitating conditions are positively related to physicians’ intentions to provide their patients with tailored health information via a digital service. However, there was no relation between facilitating condition (β=−.01; *P*=.77) and physicians’ providing intentions. Thus, H4 had to be rejected.

H5 concerned the monetary costs that are associated with the provision of a DHIS, which we assumed to be positively related to physicians’ intentions to provide such a service. However, price value (β=.03; *P*=.46) did not serve as a facilitating factor of physicians’ intentions. Therefore, H5 had to be rejected.

H6 focused on the habit of technology use being positively related to physicians’ intentions. Our results indicated that habit (β=.05; *P*=.33) is not associated with physicians’ intentions to provide a DHIS to their patients. Accordingly, H6 was rejected.

In H7, we postulated that physicians’ personal innovativeness is related to higher intentions to provide a DHIS. Indeed, personal innovativeness (β=.25; *P*<.001) was related to the intention to provide such a service, indicating that a more positive attitude toward digital innovation tends to be a facilitating factor. Thus, H7 was supported.

## Discussion

### Principal Findings

The current study examined physicians’ intentions to provide a tailored DHIS for their patients. Our findings revealed that the physicians surveyed showed moderate intentions, with the majority (n=197, 54.1%) being rather willing to provide such a service. Guided by the theoretical framework of the UTAUT2, we identified factors that are associated with their intentions. Perceived usefulness for job performance, perceived usefulness for patients, and personal innovativeness were positively associated with their intentions to provide a DHIS, while perceived ease of use, social influence, facilitating conditions, price value, and habit were not significantly associated with their intentions.

### Theoretical Implications for a Model of Physicians’ Intentions to Provide a DHIS

The analysis revealed that the tested model, based on the UTAUT2, accounted for 56.8% of the variance. Nevertheless, the effectiveness of this framework in explaining physicians’ intentions to provide a DHIS is questionable, as only 3 out of the 9 integrated factors contributed significantly to variance explanation. Physicians’ perceived usefulness for job performance and perceived usefulness for patients were most strongly associated with their intentions to provide a DHIS, which is in line with prior research on health professionals’ intentions to use health-related technology in general [18,19,28].

Furthermore, the inclusion of personal innovativeness within the UTAUT2 framework demonstrated a positive association with physicians’ intentions to provide a DHIS. This finding strengthens the suggestion to integrate the personal innovativeness construct into models explaining physicians’ intentions to engage with digital services. Such an implication is also supported in prior studies on both patients’ [33,34] and physicians’ [37] acceptance of digital health technologies. As an interim conclusion, it can therefore be stated that performance expectancy, separated for job performance and patient outcomes, and personal innovativeness could serve as basic factors for a robust model.

The majority of theoretically derived factors were not associated with physicians’ intentions to provide a tailored DHIS. First, perceived ease of use did not prove to be relevant, indicating that in the specific context of a tailored DHIS, its implementation might be worth any effort, as digital health information is a “common good” [10] that should be accessible to everyone [11]. In this regard, Zhai et al [39] point out that physicians usually have a high workload and therefore might not mind spending more time on familiarization with a new service that could diminish their workload. In addition, the fact that perceived ease of use is not a key determinant of physicians’ intentions could be related to the sample of the online access panel, which may be more digitally savvy and therefore perceive fewer difficulties in the context of handling DHIS. The potential value of integrating perceived ease of use into a model for explaining physicians’ intentions to provide a DHIS should thus be explored with a less digitally savvy sample.

Second, social influence—the perception that patients or colleagues think one should provide a DHIS—was irrelevant to the physicians’ intentions. However, the mean scores of these variables were mediocre, indicating that norms regarding the provision of a DHIS might not be established and thus be less influential. Indeed, digital health services are not yet widely used in Germany [43]. In this regard, our study found that only 15.4% (n=56) of respondents had already provided a comparable service to their patients. The inclusion of social influence as a potential determinant of physicians’ intention to provide a DHIS could therefore be fruitful at a later stage, when the relevant norms are more established in Germany.

In addition, facilitating conditions were not related to physicians’ intentions to provide a DHIS. This finding suggests that having the necessary infrastructure such as technical resources and support might not be relevant until the actual implementation of such a service, which is in line with the first version of the UTAUT framework [24]. Moreover, physicians may already have a good infrastructure in this respect. For example, a DHIS only requires computer and internet access, which is available in the majority of German practices and hospitals. In addition, the presumably digitally savvy participants of an online access panel may also have the technical know-how to use a DHIS. Therefore, it may be worthwhile to investigate the significance of facilitating conditions within a less digitally savvy sample, while also considering the digital infrastructure of the respective medical facilities.

Furthermore, price value, as measured in this study, was not related to physicians’ intentions to provide a DHIS. The item used reflected physicians’ preference for the service to be free of charge, rather than the traditional UTAUT2 definition of price value, which captures the trade-off between perceived benefits and costs. While many physicians indicated that the service should be free of charge, this does not allow us to conclude the influence of actual costs on their intention to provide a DHIS. Previous studies often did not include price value when examining usage intentions of health-related technologies, as it may be less relevant in professional health care contexts [27,31,33,38]. This highlights the need for context-specific adaptations when specifying models to explain physicians’ intentions to use digital health technologies.

Habit was also not related to physicians’ intentions to provide a DHIS. Thus, familiarity with digital technologies in general is not relevant in the context of a DHIS. This result could be attributed to our measurement. As DHIS are not yet widely used in Germany [43], we asked about physicians’ habits regarding digital technologies in general and not about the perceived routine of using a DHIS in particular. Thus, integrating habit in handling a DHIS as a determinant of physicians’ intentions to provide such a service appears less meaningful until DHIS are more established in Germany.

### Limitations and Resulting Tasks for Future Research

Our study is not without limitations. First, as we conducted a cross-sectional survey, it is not possible to draw causal conclusions. Second, our study is not representative of all physicians in Germany. Since we focused on physicians working in practices and did not stratify by their expertise, physicians in hospitals and other medical institutions as well as certain specialties are underrepresented in this study. Hence, a potential bias may exist, given that a DHIS may be considered more beneficial for certain disciplines. In addition, the majority of the surveyed physicians were participants in an online access panel and are possibly more digitally savvy, which does not necessarily reflect the perspective of most physicians in Germany.

Third, the physicians assessed their intentions to provide a DHIS solely based on a textual description of its features, which introduced the service in general terms and presented it as easy to use. They did not have the opportunity to try it out with their patients, and item-level responses may reflect assumptions based on the vignette rather than experience with a system. As a result, intentions reported in this study may not fully correspond to the actual provision of a DHIS. Although this approach is common in survey-based technology acceptance research, it may limit the validity of our findings. Future research should test a DHIS in practice, focusing on specific specialties to improve the quality of usage prediction by evaluating the actual use in a particular context (eg, cardiology) rather than intentions in general. Experimental or longitudinal designs could be used to allow for causal inferences and to explore the effectiveness of such a service on patient outcomes and physicians’ job performance.

A fourth limitation concerns the theoretical framework applied in this study. Although UTAUT2 has been widely used in health care research, it is a consumer-oriented acceptance model that may not fully capture the professional and organizational context of physicians as users of digital health technologies. We partly addressed this limitation by adapting the model to the study context, for example, by excluding hedonic motivation. Nevertheless, future research should consider extending acceptance models to more explicitly reflect physicians’ professional roles and decision-making contexts. Although hedonic motivation was excluded based on theoretical and contextual considerations, future studies could empirically examine its relevance in professional health care settings to determine whether enjoyment contributes to physicians’ acceptance of DHIS.

### Practical Implications

There are several practical implications that can be derived from our findings. First, when promoting a DHIS to physicians, the personal value for improving their daily work life, patient communication, and patient outcomes should be addressed. In detail, it should be explained that a DHIS can save time during the medical consultation and has great potential to support patients’ decision-making by providing important, tailored health information. Second, physicians with a positive attitude toward digital innovation may support dissemination by sharing experiences and demonstrating use cases at medical congresses or other professional events, thereby increasing awareness of DHIS options. In addition, a digital information service and its potential could be presented in national medical journals, which could contribute to the establishment of social norms. Another sustainable approach to promote the provision of digital health information is the implementation of eHealth in professional education. At last, in the sense of empowerment and patient-centered care, patients could be encouraged to claim their right to be adequately informed and to directly ask their physicians for digital health information. Furthermore, the effects of providing a tailored DHIS on patient outcomes need to be examined in more detail to provide comprehensive evidence.

### Conclusions

In conclusion, the perspective of the majority of surveyed physicians suggests that a tailored DHIS seems to be a promising way to provide additional health information and thus enhance face-to-face physician-patient communication. The key determinants of physicians’ intentions to provide such a service—perceived usefulness for improving job performance and patient outcomes as well as physicians’ personal innovativeness—underscore the importance of addressing these aspects when supporting its implementation. Future research should aim to identify additional determinants to further advance the development of a more comprehensive model and focus efforts to establish DHIS in Germany.

## Supplementary material

10.2196/80620 Multimedia Appendix 1 Introduction of the digital health information service and survey items.

## Abbreviations

DHIS: digital health information service
UTAUT: unified theory of acceptance and use of technology
UTAUT2: extended unified theory of acceptance and use of technology
