# Supplementary material for: Physician-Patient Communication and Physicians’ Acceptance of a Tailored Digital Health Information Service: Quantitative Online Survey
Source: JMIR Hum Factors. 2026 Mar 6;13:e80620. doi: 10.2196/80620 (PMC12978895; doi:10.2196/80620)
Supplement: Multimedia Appendix 1 [file humanfactors-v13-e80620-s001.docx]

### S1. Introduction of the DHIS

The following is specifically about tailored digital information services. Please read the following text carefully.

Imagine that you had the opportunity to provide your patients with the most important information about their diseases and treatments, in addition to your explanations during the consultation, in an uncomplicated and individual way via a tailored digital information service.

With such a tailored health information service, you as a physician could use a digital web-based platform to select the information your patient needs in a specific situation (e.g., on the basics of a disease, symptoms, risks, or treatment options). Your patients could access this information, which is tailored to them personally, at their leisure from home via a computer or smartphone.

In the following, we are interested in your opinion, expectations and requirements regarding such a tailored digital information service.

### S2. Survey Items

| **Construct** | **Items** | **Source** |
| --- | --- | --- |
| Intention to provide a tailored DHIS | *I1.* I intend to (continue to) provide a tailored digital information service for my patients in the future.  *I2.* I think I would (continue to) provide a tailored digital information service for my patients in the future.  *I3.* I am interested (in continuing) to provide a tailored digital information service for my patients. | Venkatesh et al., 2012 |
| Usefulness for job performance | *PEJ1.* Providing a tailored digital information service is useful in my daily work life.  *PEJ2.* A tailored digital information service saves me a lot of time during office hours.  *PEJ3.* A tailored digital information service improves communication with my patients.  *PEJ4.* A tailored digital information service enables individualized advice for my patients.  *PEJ5.* With the help of a tailored digital information service, I can better engage my patients in health-related decisions. | Venkatesh et al., 2012 |
| Usefulness for patients | *PEP1.* A tailored digital information service is useful for my patients.  *PEP2.* A tailored digital information service can help my patients to make health decisions more quickly.  *PEP3.* Providing a tailored digital information service helps to meet my patients’ information needs.  *PEP4.* With the help of tailored digital information service, my patients are better able to maintain or improve their health.  *PEP5.* A tailored digital information service can contribute to the empowerment of my patients. | Venkatesh et al., 2012 |
| Ease of use | *EEU1.* Learning how to use a tailored digital information service is easy for me.  *EEU2.* My interaction with a tailored digital information service is clear and understandable.  *EEU3.* Providing such a tailored digital information service to my patients is easy for me. | Venkatesh et al., 2012 |
| Subjective norm (patients) | *SNP1.* Most of my patients think that I should provide a tailored digital information service for them.  *SNP2.* Most of my patients use digital information services.  *SNP3.* Most of my patients would appreciate me providing a tailored digital information experience for them. | Park & Smith, 2007; Venkatesh et al., 2012 |
| Subjective norm (colleagues) | *SNC1.* Most of my colleagues think that one should provide digital information to one’s patients.  *SNC2.* Most of my colleagues would provide their patients with a tailored digital information service.  *SNC3.* Most of my colleagues appreciate providing patients with as much information as possible. | Park & Smith, 2007; Venkatesh et al., 2012 |
| Facilitating conditions | *FC1.* I have the necessary technical equipment in my consulting room to provide a tailored digital information service (e.g., computer, Internet access).  *FC2.* I have the necessary technical knowledge to provide my patients with a tailored digital information service.  *FC3.* A tailored digital information service is compatible with other digital applications I use in my practice/hospital.  *FC4.* I can get help from others when I have difficulties using a tailored digital information service. | Venkatesh et al., 2012 |
| Price value | *PV1.* The use of a tailored digital information service should be free of charge for me.  *PV2.* The use of a tailored digital information service should be free of charge for my patients. *(dropped)*  *PV3.* It should be possible to account for the provision of a tailored digital information service. *(dropped)* | Damschroder et al., 2009; Venkatesh et al., 2012 |
| Habit | *HT1.* The use of digital technologies has become a habit for me.  *HT2.* I frequently use digital technologies in my daily work life.  *HT3.* It is important to me to integrate digital services into my daily work life. | Venkatesh et al., 2012 |
| Personal innovativeness | *PI1*. I have a positive attitude toward digital innovations.  *PI2.* When I hear about a new technology or digital service, I want to try it out.  *PI3.* In my environment, I am usually one of the first to try out new technologies. | Baudier et al., 2021 |
